# A Frailty Index based on clinical data to quantify mortality risk in dogs

**DOI:** 10.1038/s41598-019-52585-9

**Published:** 2019-11-14

**Authors:** Tommaso Banzato, Giovanni Franzo, Roberta Di Maggio, Elisa Nicoletto, Silvia Burti, Matteo Cesari, Marco Canevelli

**Affiliations:** 1 Department of Animal Medicine, Productions and Health, University of Padua, Viale dell’Università 16, Legnaro, Italy; 2 Fondazione IRCCS Ca’ Granda Ospedale Maggiore Policlinico, Milano, Italy; 3 Geriatric Unit, Department of Clinical Sciences and Community Health, University of Milan, Milano, Italy; 4 Department of Human Neuroscience, Sapienza University, Rome, Italy

**Keywords:** Prognostic markers, Geriatrics

## Abstract

Frailty is defined as a decline in an organism’s physiological reserves resulting in increased vulnerability to stressors. In humans, a single continuous variable, the so-called Frailty Index (FI), can be obtained by multidimensionally assessing the biological complexity of an ageing organism. Here, we evaluate this variability in dogs and compare it to the data available for humans. In dogs, there was a moderate correlation between age and the FI, and the distribution of the FI increased with age. Deficit accumulation was strongly related to mortality. The effect of age, when combined with the FI, was negligible. No sex-related differences were evident. The FI could be considered in epidemiological studies and/or experimental trials to account for the potential confounding effects of the health status of individual dogs. The age-related deficit accumulation reported in dogs is similar to that demonstrated in humans. Therefore, dogs might represent an excellent model for human aging studies.

## Introduction

In recent decades, the quality of veterinary care has markedly increased, reaching, in several fields, the standards of human medicine^1^. Indeed, complex diagnostic examinations, such as radiology, ultrasonography, blood testing, computed tomography and magnetic resonance imaging, that were previously available only for human patients are currently commonly performed as part of the routine clinical evaluation of our companion animals. In parallel, relevant advances in therapeutic options have been achieved, resulting in the improved management of numerous diseases, leading to an increase in the life expectancy of domestic dogs and cats^2^. As a consequence, the management of geriatric patients is becoming an important challenge for veterinary medicine^3^. Nevertheless, only a limited amount of evidence-based studies focusing on the different aspects of clinical care^3–5^ of older veterinary patients are currently available.

Although the field of geriatrics is well established, traditional medical models can be inadequate when applied to age-related pathological conditions. Paradigms, such as chronological age and individual disease entities, do not fully capture an older person’s clinical/biological complexity, risk profile and clinical needs. Accordingly, this data may be inappropriate to inform and sustain medical choices and interventions. These considerations can likely be extended to any ageing animal, regardless of species.

Based on these premises, the concept of frailty is receiving increasing attention in the scientific community. Frailty is defined as a decline in an organism’s physiological reserves resulting in increased vulnerability to stressors. It accounts for a wide variability in health outcomes and trajectories among living beings of the same chronological age^6^. It is, in fact, a common experience that not only people but also animals of the same age have the same risk of death and other adverse outcomes^7^. The frailty status of an organism can be measured by counting the negative health attributes presented by the individual over the life course^6,8–12^. A single continuous variable, the so-called Frailty Index (FI), can be obtained by multidimensionally and comprehensively assessing the ageing organism. It is noteworthy that in addition to being widely adopted to stratify risk profiles and support decision-making in various fields of human medicine, the FI has already been used in laboratory animals (i.e., mice and rats), showing robust predictive capacity^8,13–16^.

Unfortunately, frailty is still a relatively neglected concept in veterinary medicine. For example, only a single attempt to quantify and measure frailty in dogs has been made^17^. Clinical decisions are still mostly based on traditional models of care considering mono-dimensional approaches, and no attempt to comprehensively evaluate the complexity of the whole system is made. It can be hypothesized that the deficit accumulation model may enhance and refine the clinical approach and the prognostic capacity in dogs during ageing. Moreover, the FI could be used in experimental and epidemiological studies to account for the potential confounding effects of age-related conditions. Therefore, the aims of the present study are as follows: 1) to develop a FI in dogs based on routinely collected clinical variables; 2) to assess the accuracy of this newly developed FI in the prediction of short-term mortality; and 3) to compare the results obtained in dogs with the results obtained in humans.

## Results

### Dog population

**Figure 1:**
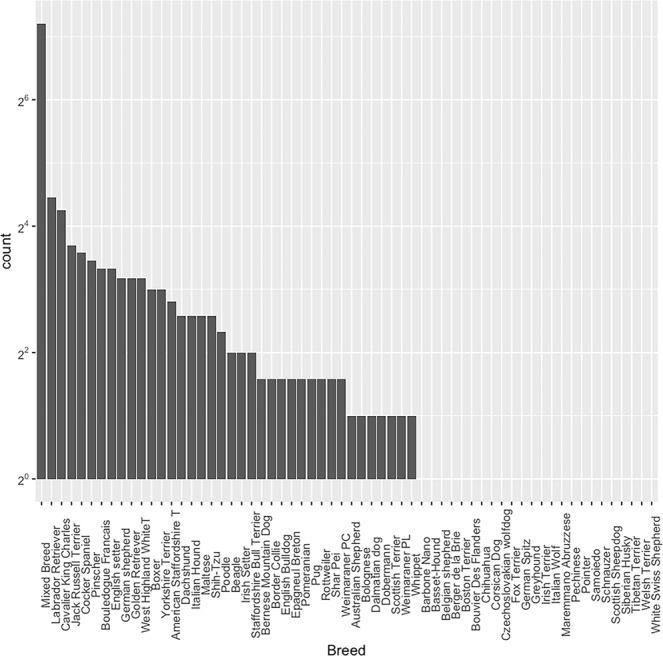
Number of dogs (reported as log 2 of the total number) for each breed included in the study.

A total of 401 dogs of various breeds met the inclusion criteria and were considered for the present analysis. The mean age was 8.64 (range = 2–19) years. Of the 401 dogs, 75 were intact females (mean age 8.37, range 2–16, years), 142 were neutered females (mean age 9.23, range 2–19, years), 147 were intact males (mean age 8.31, range 2–16, years), and 37 were neutered males (mean age 8.27 range 2–16, years). A list of the breeds of the dogs along with the number of individuals belonging to each breed is reported in Fig. 1.

### The distribution of the FI

**Figure 2:**
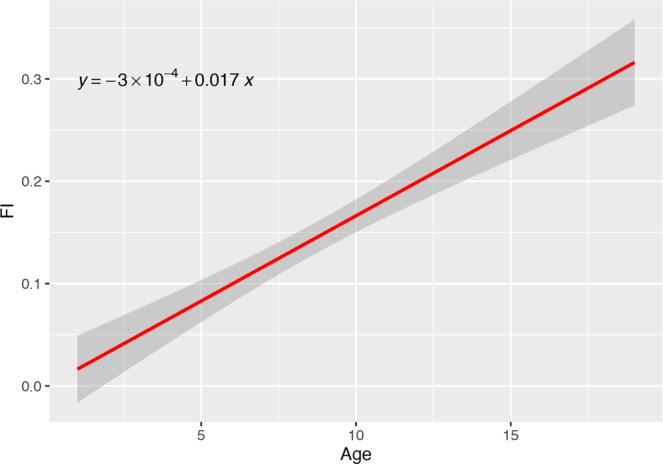
Plot of the Frailty Index according to age. The red line is the regression line.

**Figure 3:**
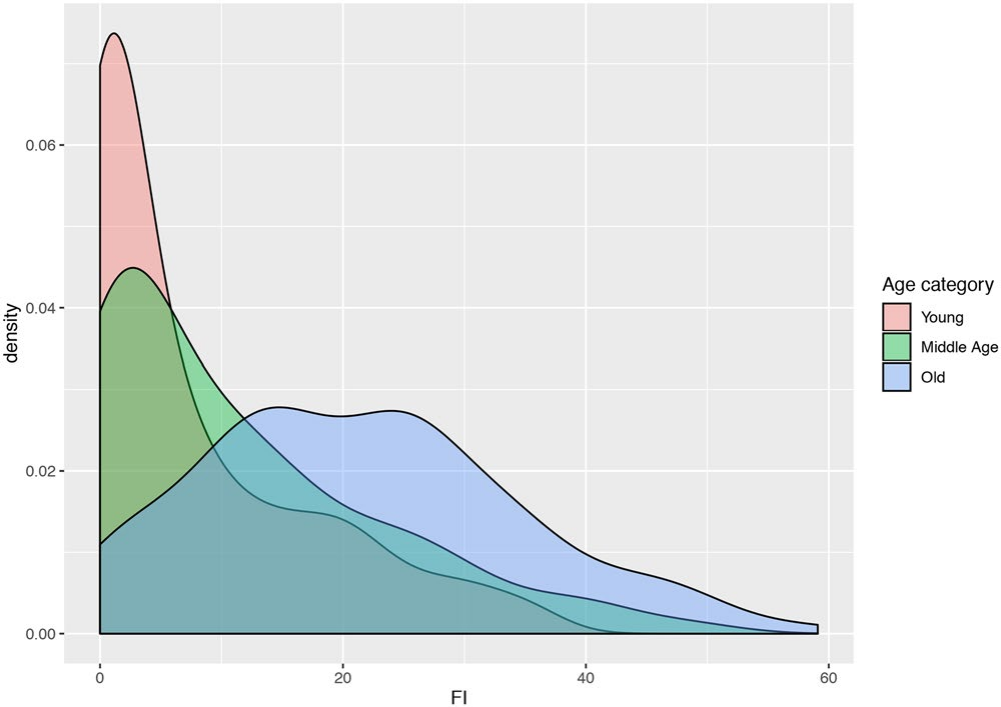
Density plot of the FI in the three age categories: young dogs (2–6 years), middle-aged (7–10 years), old dogs (10+ years).

The mean FI in our dog population was 0.14 (SD 0.13, interval 0.0–0.61). A regression plot depicting the FI in relation to age, along with the regression line, is reported in Fig. 2. The mean FI increased from 0.08 (SD 0.1, interval 0–0.36) in young dogs (n = 137) to 0.11 (SD 0.12, interval 0–0.48) in middle-aged dogs (n = 114) to 0.23 (SD 0.13, interval 0–0.61) in old dogs (n = 150). The summary statistics of the FI are reported in Table 1. A density plot showing the distribution of the FI according to age is reported in Fig. 3. Overall, a significant positive correlation, which followed an essentially linear relationship (b = 0.016; p-value < 0.001), was observed between age (in years) and FI value (Spearman rho = 0.51, p-value < 0.001) (Fig. 2). An increase in FI heterogeneity was observed in older animals (studentized Breusch-Pagan test, p-value = <0.001).

**Table 1:** Summary statistics of the FI by age.

	Overall	Young Dogs (2–6 years)	Middle-aged dogs (7–10 years)	Old dogs (10+ years)
N	401	137	114	150
Mean FI	0.14	0.08	0.11	0.23
Interval	0–0.61	0–0.36	0–0.48	0–0.61
99^th^ percentile	0.59	0.35	0.48	0.61

### The FI showed moderate accuracy in the prediction of short-term mortality

**Figure 4:**
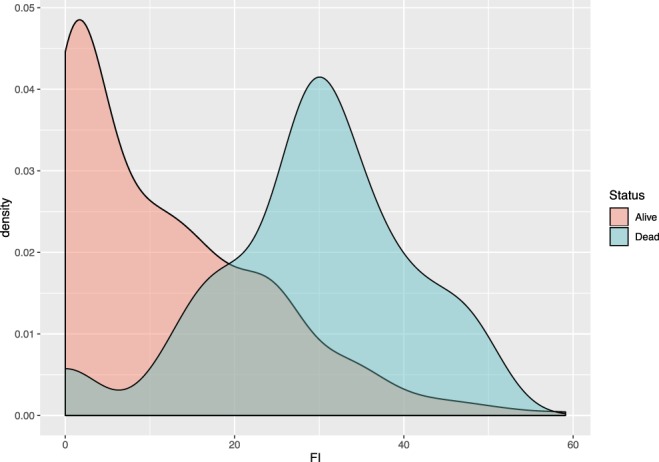
Density plot of the Frailty Index in dogs that survived and in dogs that died during the observation period.

**Figure 5:**
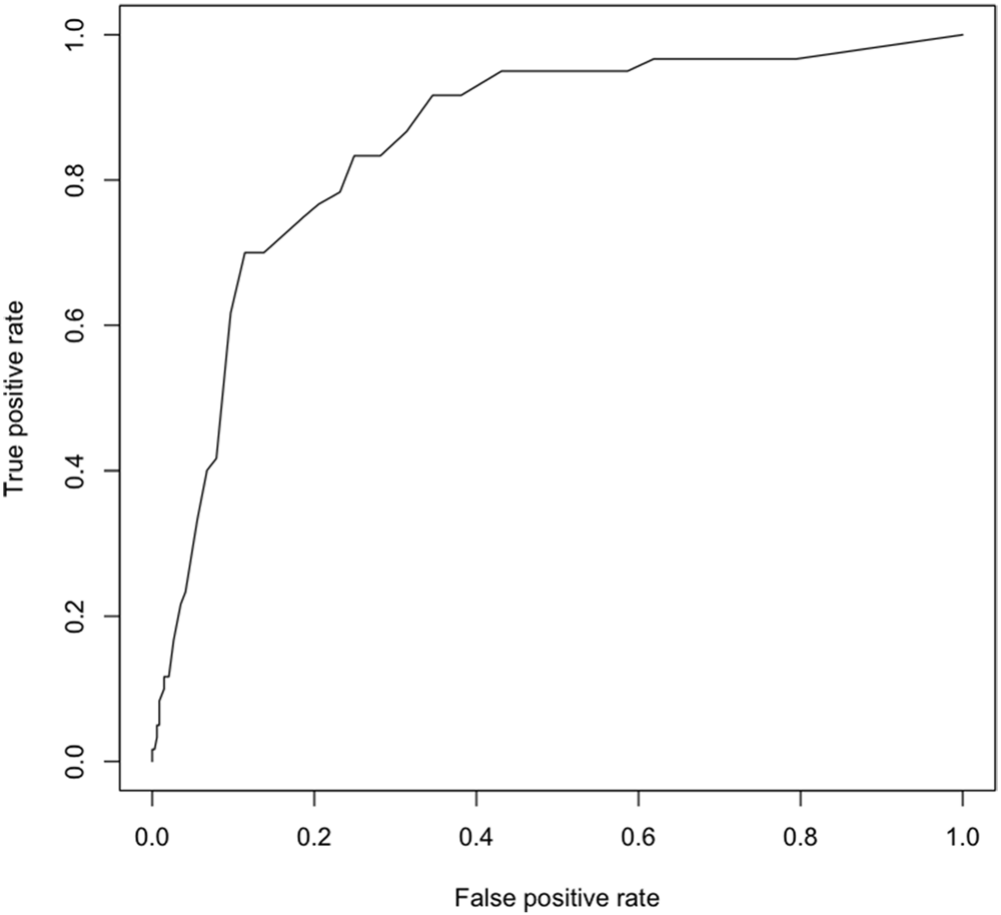
Receiver operator characteristic curve of the FI in the prediction of short-term mortality (six months). Considering a cut-off value of 0.25, the area under the curve was 0.852 (95% confidence interval (CI) = 0.814–0.885), the sensitivity was 70.0% (95% CI 56.8–81.2), the specificity was 88.56 (95% CI 84.7–91.7), the positive predictive value was 6.12 (95% CI 4.4–8.6), and the negative predictive value was 0.34 (95% CI 0.2–0.5).

The density plot of the FI in dogs that survived or died during the observation period is reported in Fig. 4. The mean FI of the surviving dogs was 0.12 (interval 0–0.59), whereas the mean FI of the dogs that died was 0.29 (interval 0–0.48). The accuracy of the FI to predict short-term mortality was classified as moderate (Fig. 5). Using a cut-off value of 0.25, the area under the curve was 0.852 (95% confidence interval (CI) = 0.814–0.885), the sensitivity was 70.0% (95% CI 56.8–81.2), the specificity was 88.56 (95% CI 84.7–91.7), the positive likelihood ratio value was 6.12 (95% CI 4.4–8.6), and the negative likelihood ratio was 0.34 (95% CI 0.2–0.5). The complete results of the ROC curve analysis are reported in Annex 2.

### Deficit accumulation is related to increased mortality risk in dogs

**Figure 6:**
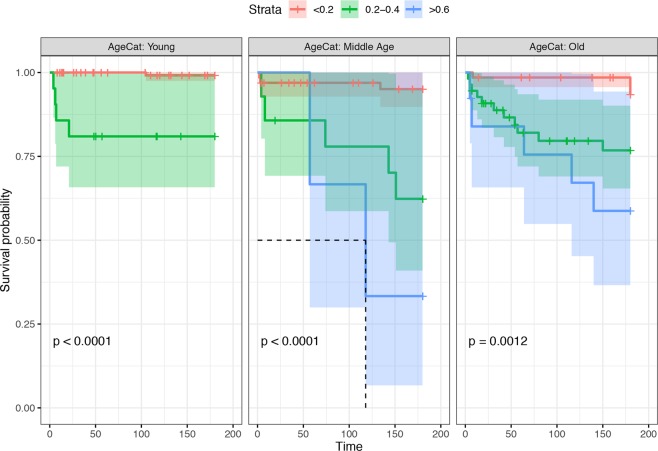
Kaplan-Meier survival curves stratified by age and 0.2 increments of the FI. The Kaplan-Meier survival curves of dogs classified in different FIs (colour coded) and age (different panels) categories are reported. Censoring events are indicated as vertical dashes. The survival median line and the 95% confidence intervals (shaded areas) are also provided.

**Figure 7:**
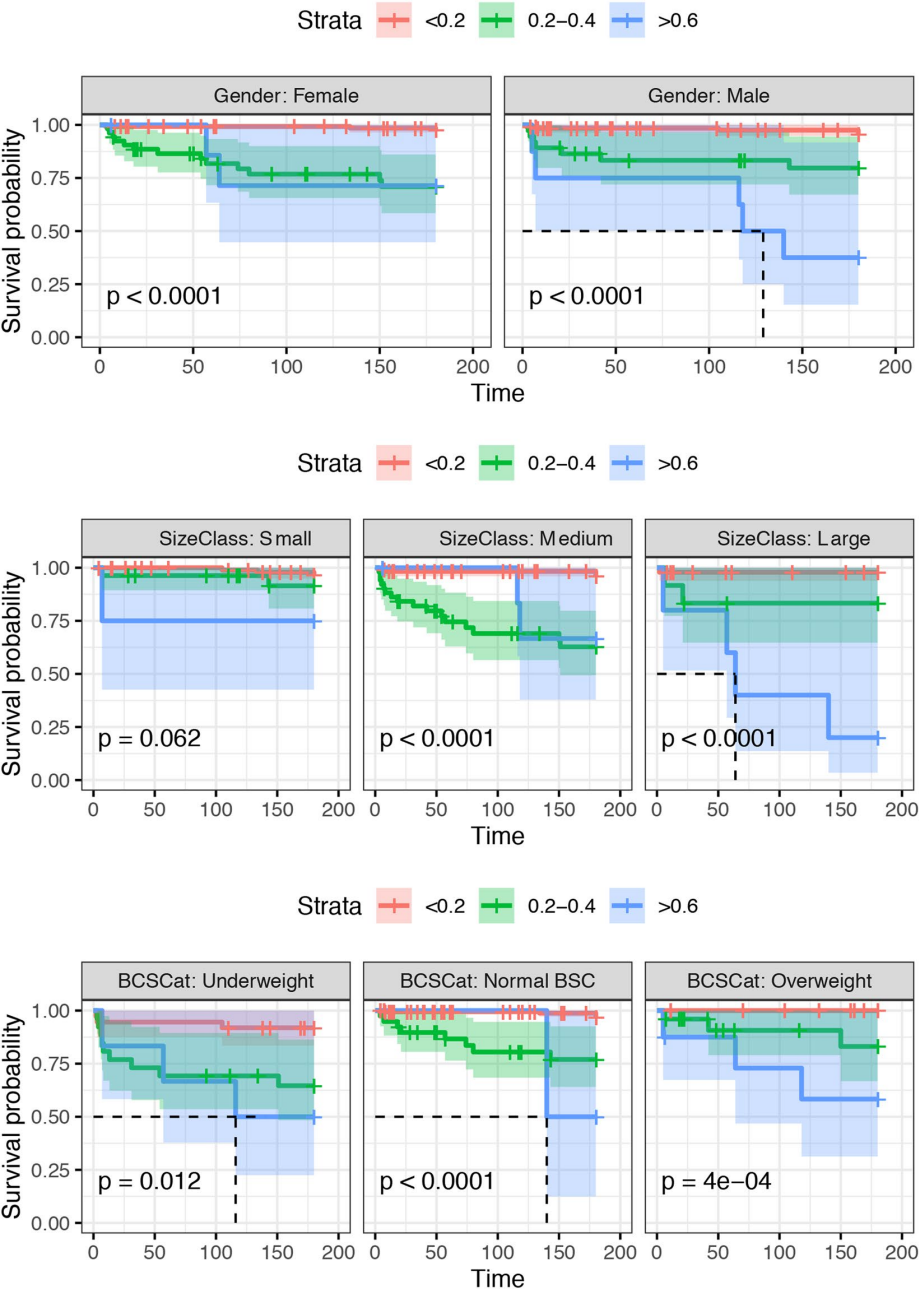
Kaplan-Meier survival curves stratified by age and 0.2 increments of the FI. The Kaplan-Meier survival curves of dogs classified in different FIs (colour coded) and gender (different panels), size class (classified as small dogs <10 kg, medium sized dogs ≤10–<30 kg, and large dogs ≥ 30 kg), and body condition score (BCS) (underweight (BCS < 5), normal (5 ≤ BCS ≤ 6) and overweight (BCS > 6) categories are reported. Censoring events are indicated as vertical dashes. The survival median line and the 95% confidence intervals (shaded areas) are also provided.

The log-rank test comparing the Kaplan-Meier curves showed a significant difference in 6-month survival probabilities according to the FI category, both in the overall analysis (p-value < 0.001) and in the age (Fig. 6), sex, BCS and size category stratified analyses (p-value < 0.001) (Fig. 7 and Annex 3). Cox proportional hazards regression demonstrated a significant effect of the FI when it was considered a continuous variable (hazard Ratio (HR) = 1.08 [95% CI = 1.06–1.10], p-value < 0.001) and a discretized variable (0.2 < FI < 0.4 HR = 9.21 (95% CI = 4.05–20.96), p-value < 0.001; FI > 0.4 HR = 18.06 (95% CI = 6.54–49.88), p-value < 0.001). While animal age showed a significant effect on HR when considered univariately (HR = 1.16 (95% CI = 1.07–1.27), p-value < 0.001), it did not remain significant when it was combined with the FI (p-value = 0.343), and its inclusion did not lead to a significant improvement in the prediction of the model. Similarly, no effect of sex (p-value = 0.634) was detected. However, the FI was still statistically significant (p < 0.001) when both sex and age were included in the model.

When the BCS was considered as a covariate of the FI category, an increased BCS acted as a protective effect. In particular, the hazard ratio (i.e., HR = 0.38; p-value = 0.049) of overweight dogs was less than half that of underweight dogs. Similarly, the hazard ratio was lower in normal BCS dogs than in underweight dogs, although the difference was not statistically significant (p-value = 0.075). The size class analysis demonstrated a higher hazard ratio in middle-sized dogs (HR = 2.6650; p-value = 0.033) than in small-sized dogs. Similarly, large breeds demonstrated an increased risk (HR = 2.407), although it was not statistically significant (p-value = 0.116). In all cases, the FI remained statistically significant (P < 0.001) (Fig. 7 and Annex 3). Identical results were obtained when both the size category and BCS were included in the final model (Fig. 7 and Annex 3).

## Discussion

To our knowledge, this is the first study to develop and implement a clinical-based FI in dogs. Overall, the index exhibited a moderate accuracy in the stratification of mortality-risk profiles over the short term. The health deficits that were included in the questionnaire were chosen to closely resemble the data that are routinely collected during clinical evaluations of dogs. This choice was motivated by the attempt to show how easy is to reproduce this quantitative (not qualitative) instrument. In fact, the adopted model of frailty is based on the assumption that the predictive capacity of the instrument is not attributable to specific items but by the accumulation of information (stemming from a multidimensional assessment of the organism)^11^. Considering this perspective, the data routinely collected during a clinical assessment represents an ideal data set from which the index can be computed.

In humans, FIs based on the clinical data of the patient^11^ and on laboratory evaluations (mainly blood tests) have been developed^8,18–20^. An FI combining both clinical and laboratory findings has been developed in mice^21^. In this study, we decided to include only the subjects who had recent blood tests for two main reasons 1) thoroughly assessing the mental status of a dog, except for in obvious situations, is difficult and requires a specialty neurological examination. Therefore, we decided to focus our questionnaire mainly on the physical and laboratory findings. 2) It has been reported that both physical and laboratory abnormalities are often present in apparently healthy elderly dogs^3^. Given the limited information that can be obtained from clinical evaluations of dogs, we felt that establishing strict inclusion criterion could provide an in-depth and reliable evaluation of the subjects. On the other hand, based on this selection procedure, our study population might not be fully representative of the general dog population. Indeed, blood examinations are not routinely performed on healthy animals; therefore, their inclusion might have captured deficits with limited clinical relevance. As a consequence, the mean FI of the subjects included in this study might have been overestimated. A standard procedure to calculate the frailty index based on only a clinical evaluation of the subjects has also been proposed in mice^15^.

The age categories of the dogs were created to closely resemble those that are commonly used to describe the distribution of the FI in humans, and interestingly, the age-stratified mean FIs in dogs showed similarities with the mean FIs reported in humans^8^. Indeed, the mean FI values reported in a large (n = 9.169) representative sample of young (20–44 years), middle-aged (45–64 years), and old (65+ years) individuals were 0.08 (SD 0.07), 0.16 (SD 0.11), and 0.24 (SD 0.13), respectively. Interestingly, the mean FI calculated in our overall dog population (i.e., 0.14, SD 0.13) was similar to the overall FI in community-dwelling individuals (i.e., 0.15, SD 0.12)^8^. Another interesting similarity is that the coefficient of variation of the FI decreased with age in dogs, a feature that is also typical in humans; indeed, both the average FI and the variation increased with age^7^. Moreover, as commonly reported in humans, the FI showed a submaximal limit at the 0.7 cut-off point, which is traditionally considered as the threshold when the accumulation of deficits becomes incompatible with life.

Several studies^1,22–24^ suggest that dogs serve as a very good model to study ageing and age-related diseases. In particular, recent studies have highlighted similarities in the ageing process in dogs and humans regarding the immune system^25^, the nervous system^26^, the muscular system (in particular sarcopenia)^27^, DNA methylation with age^28^, and telomere length^29^. On the other hand, a limited number of studies concerning the dog ageing process are available, and for some body systems, these studies are also quite outdated^30^. The results of this study highlight that the ageing processes in dogs and humans potentially follow similar dynamics, thus confirming a potentially central role of dogs in ageing research. On the other hand, several exceptions were evident; some dogs with a very high FI (i.e., >0.5) survived more than six months, whereas some dogs with a low FI (or even no considered deficits) died within the observation period. Similar findings have been reported in human medicine^31^. It must be emphasized that, in contrast to human beings, the follow-up period considered in the present study (i.e., 6 months) represents a significant fraction of a dog’s average life expectancy. Therefore, the factors contributing to the FI (most being chronic deficits) could have had more time to affect the dog, leading to death before censoring and increasing the FI predictive performance in the present study. Indeed, the accuracy of the FI in the prediction of short-term mortality (3 months) in a large cohort of human patients was 0.69 with a ﻿sensitivity of 23% (95% CI 22–24%) and a specificity of 91% (95% CI 91–91%)^32^.

Sex-specific differences in frailty scores have been reported in both humans^33,34^ and in mice^21,35^. In particular, in humans, the so-called ﻿“male-female health-survival paradox” has been described; females live longer than males despite having increased average FI scores at all ages^34^. Interestingly, no sex-specific differences were evident in our study. It is our opinion that the difference in the results is mainly related to the limited study population (401) compared to the large human populations (﻿37.426) included in other studies. Another difference between humans and dogs is that a remarkable percentage of the dog population is neutered, and neutering has been associated with an increase of the lifespan in dogs^36^. Such an increase is more pronounced in females (+26.3%) than in males (+13.8%). The effect of neutering was not considered here because of the limited study population and the limited number of neutered males (37/184) included in the study.

Interestingly, a protective effect of a high BCS on overall survival was evident according to the Cox regression analysis. These results confirm, for example, the reported increased survival time of overweight dogs affected by chronic kidney disease compared to dogs with a normal BCS^37^. A similar protective effect of overweight on the outcomes of some disease (e.g., heart failure, cancer, chronic obstructive pulmonary disease), sometimes referred to as “the obesity paradox”, is also reported in humans^38^.

The results of this study suggest that the FI should be routinely calculated in veterinary clinical practice, especially for older patients. The possibility of quantitatively evaluating frailty in dogs using an inexpensive tool could assist the veterinary clinician in selecting the best treatment options for each patient. The moderate accuracy of the FI in the prediction of short-term mortality in dogs suggests that patients with a high FI (>0.25) should be treated with particular care and might need more frequent follow-up visits than subjects with a low FI, regardless of their age.

A potential limitation of the present study is that the influence of breed on the relationship between frailty and age was not evaluated. This interaction was not explored because of the large number of breeds included in this study (n = 62) and because of the marked differences in the numerosity of dogs belonging to the different breeds (Fig. 1). On the other hand, the effect of size was considered in the survival analysis. Furthermore, the classification of mixed breed dogs, which were highly represented in this study (i.e., 114/401), is unclear. The prevalence of inherited defects that may potentially affect an animal’s frailty status^39^ among different breeds is well known in veterinary medicine. Moreover, it has been reported that the life span of different dog breeds is associated with several different factors, such as size, metabolic rate, and telomere length^29^. Other possible limitations of this study are that the effect of several potential confounders, such as different living environments (city vs countryside), neutering, habits of the owners (e.g., smokers vs non-smokers), level of physical activity, and others, were not evaluated. Further studies, possibly including an increased number of subjects, could help to clarify how breed might affect the relationship between frailty and mortality.

Our study shows that the FI may represent a valuable yet completely inexpensive tool to assess frailty in dogs and estimate their mortality risk. This model may provide important insights concerning the risk of age-related conditions. Additionally, the FI may be considered in epidemiological studies and/or experimental trials to account for the many potential confounders associated with a dog’s heath status. The close similarity with the FI data from human studies confirms that the dog can be a valuable model for translational research on ageing.

## Methods

### Study design and samples

Privately owned dogs admitted between October 2017 and January 2019 to the Veterinary Teaching Hospital of the University of Padua for specialty examination or for routine examination were included in the study. Complete signalment and a medical history were recorded for each patient. The inclusion criteria were as follows: 1) age ≥ 2 years; and 2) the availability of recent blood examinations (i.e., within the previous three months). All the procedures were carried out in accordance with the relevant Italian and EU legislation concerning Animal Protection and Welfare (Leg. Decree 26/2014 implementing the EU directive 2010/63/EU). Since the data used in this study were collected as part of routine clinical activity, no ethical committee approval was needed. Informed consent regarding personal data processing was obtained from the owners.

### Anamnestic data collection

Basic information, including age, sex (considering four categories: intact male, neutered male, intact female, neutered female), breed, weight, body condition score (based on the WSAVA guidelines)^40^, diet (commercial, homemade, mixed), vaccination status, dirofilariasis prophylaxis status, and hospitalization during the last year were recorded.

### Frailty index construction

The FI was calculated using a non-predefined checklist of individual health deficits. The third part of the questionnaire contained questions to address 33 health deficits that were used to calculate the FI. The choice of the deficits was based on the standard procedure suggested by Searle *et al*.^11^ and adapted to describe the specific characteristics of the canine species. In particular, each included deficit met the following criteria: 1) the deficit was adversely related to the animal’s health status; 2) the deficit was age-related (i.e., it tends to increase with age); and 3) the deficit was not prone to saturation (i.e., the deficit was neither too rare nor too frequent). Moreover, these variables were not restricted to single organs or systems, thus assuring the multidimensionality of the index. Some health deficits were assigned a score of 0 (if absent) or 1 (if present). Some other deficits were evaluated using a semi-quantitative scale as absent (score = 0), mild (score = 0.5), or severe (score = 1). The index was then calculated by dividing the sum of the health deficits of each individual by the number of deficits included in the questionnaire (i.e., 33)^10^, thus producing a total score ranging between 0 and 1. The complete questionnaire developed in this study is attached as Annex 1.

### Questionnaire collection

All the questionnaires were completed by one of the authors of this study (TB, RDM, EN, SB, with 10, 5, 2, and 2 years of clinical experience, respectively) based both on the responses of the owner during the medical history interview and the results of clinical diagnostic tests (ultrasounds, radiographs, blood examinations, etc.) performed during the clinical evaluation of the patients. Short-term (i.e., 6-month) mortality was recorded based on the subsequent assessments. If no assessment was performed after six months, the dog public registry of the Veneto Region (http://cani.crev.it/acsvr/search/) was consulted to verify the living status. Fatalities were excluded from the study. Mortality was last monitored on July 2019.

### Statistical and data analysis

The descriptive statistics of the individual health deficits in relation to age was calculated. The distribution of the FI according to age was analysed, considering three age categories (young: 2 to 6 years; middle-aged: 7 to 10 years, old: over 10 years). Density plots were created using the ggplot2 library^41^ of the R programming language. Results with a p-value less than 0.05were considered statistically significant significant.

Survival analysis was performed using the *survival* library in R. Kaplan-Meier cumulative survival curves were created by categorizing the FI into three categories (i.e., FI < 0.2, 0.2 ≤ FI < 0.4, FI ≥ 0.4), and the significance of the difference between the survival curves was assessed using the log-rank (M-H) test for the total population and the age-stratified groups to control for potential confounding effects. The effect of the FI on the hazard ratio was quantified by fitting a Cox proportional hazards regression model. In addition to the FI, the age, sex, BCS (underweight (BCS < 5), normal (5 ≤ BCS ≤ 6) and overweight (BCS > 6), and size classes (classified as small dogs < 10 kg, medium sized dogs ≤ 10–<30 kg, and large dogs ≥ 30 kg) were tested and included in the final model only if a statistically significant improvement in the model fit was demonstrated by the likelihood ratio test. The proportional hazards (PH) assumption was assessed by graphical method and using the Schoenfeld test.

The accuracy of the FI in the prediction of short-term mortality was also assessed. The cut-off points, sensitivity, specificity, positive predictive value, negative predictive value, and the area under the curve (AUC) of the quantitative variables showing statistical significance were analysed using receiver operating characteristic (ROC) curves. The ROC curve analysis was performed using the PRROC^42^ package in R. All the statistical analyses were performed with the R programming language which is a language and environment for statistical computing (R Foundation for Statistical Computing, Vienna, Austria, URL http://www.R-project.org/).

## Supplementary information

Questionnaire used to calculate the frailty index

Complete results of the ROC analysis

Complete results of the survival analysis

## Data Availability

Materials are available upon request from Tommaso Banzato – tommaso.banzato@unipd.it.
