# Supplementary material for: A Frailty Index based on clinical data to quantify mortality risk in dogs
Source: Sci Rep. 2019 Nov 14;9:16749. doi: 10.1038/s41598-019-52585-9 (PMC6856105; doi:10.1038/s41598-019-52585-9)
Supplement: Supplementary file 1 — Questionnaire used to calculate the frailty index [file 41598_2019_52585_MOESM1_ESM.pdf]

A Frailty Index based on clinical data to quantify mortality risk in dogs

Tommaso Banzato[1*], Giovanni Franzo[1], Roberta Di Maggio[1], Elisa Nicoletto[1], Silvia Burti[1], Matteo Cesari[2,3], Marco Canevelli[4]

Questionnaire used to calculate the frailty index

Date ________________

Owner: __________________	Phone___________________	Name of the animal: __________
Breed ______________________	Sex: F FN M MN	Date of birth: _____________
Weight: _________	Body condition score: __________	

♦ Diet	Commercial		Home made	Mixed

				Notes
♦ Up to date with vaccinations	Yes	No		______________
♦ Regular Dirofilariasis Prophylaxis	Yes	No		______________
♦ Antiparasitic treatment	Yes	No		______________
♦ Hospitalization in the last year	Yes	No		______________
1. Assistance for standing up	Yes	No		______________
2. Decreased appetite	Yes	No		______________
3. Assistance for eating	Yes	No		______________
4. Incontinence	Yes	No		______________
5. Assistance for climbing stairs	Yes	No		______________
6. Decreased activity last year	Yes	No		______________
7. Reduced cognitive ability	Yes	No		______________
8. Reduced vitality last year	Yes	No		______________
9. Weakness during exercise	Yes	No		
10. Congenital defects	Yes	No		______________
11. Weight loss (no diet or exercise)	Yes	No		______________
12. Hair opacity	Yes	No		______________
13. Chronic Therapies	Yes	No		______________
14. Epilepsy	Yes	No		______________
15. Episodes of disorientation	Yes	No		______________
16. Chronic infective disease	Yes	No		______________
17. Endocrine disease	Yes	No		______________
18. Chronic inflammation	Yes	No		______________
19. Acute vascular problems	Yes	No		______________
20. Cancer	Yes	No		______________
21. Diabetes	Yes	No		______________
22. Osteoarthrosis	No	Mild	Severe	______________
23. Hearing impairment	No	Mild	Severe	______________
24. Cardiopathy	No	Mild	Severe	______________
25. Chronic respiratory disease	No	Mild	Severe	______________
26. Hepatopathy	No	Mild	Severe	______________
27. Neurological deficits	No	Mild	Severe	______________
28. Diseases of the oral cavity	No	Mild	Severe	______________
29. Visual impairment	No	Mild	Severe	______________
30. Chronic digestive disease	No	Mild	Severe	______________
31. Disease of the Hematopoietic system	No	Mild	Severe	______________
32. Dermatological diseases	No	Mild	Severe	______________
33. Chronic kidney disease	No	Mild	Severe	______________

Only items numbered from 1 to 33 are used for the calculation of the Frailty Index. A score of 0 (No) or 1 (Yes) is assigned to items 1-21. A score of 0 (No), 0.5 (Mild), or 1 (Severe) is assigned to items 22-33. The Frailty Index was calculated in this way: $\sum$ score / 33.
