# Supplementary material for: A Frailty Index based on clinical data to quantify mortality risk in dogs
Source: Sci Rep. 2019 Nov 14;9:16749. doi: 10.1038/s41598-019-52585-9 (PMC6856105; doi:10.1038/s41598-019-52585-9)
Supplement: Supplementary file 2 — Complete results of the ROC analysis [file 41598_2019_52585_MOESM2_ESM.pdf]

A Frailty Index based on clinical data to quantify mortality risk in dogs

Tommaso Banzato[1*], Giovanni Franzo[1], Roberta Di Maggio[1], Elisa Nicoletto[1], Silvia Burti[1], Matteo Cesari[2,3], Marco Canevelli[4]

Complete results of the ROC analysis

Appendix 2. Complete results of the Receiver Operator Characteristic Curve of the FI in the prediction of short-term mortality.

Criterion	Sensitivity	95% CI	Specificity	95% CI	+LR	95% CI	-LR	95% CI
≥0	100.00	94.0 - 100.0	0.00	0.0 - 1.1	1.00	1.0 - 1.0		
>0	96.67	88.5 - 99.6	20.53	16.4 - 25.2	1.22	1.1 - 1.3	0.16	0.04 - 0.6
>0.015151515	96.67	88.5 - 99.6	31.09	26.2 - 36.3	1.40	1.3 - 1.5	0.11	0.03 - 0.4
>0.03030303	96.67	88.5 - 99.6	38.12	32.9 - 43.5	1.56	1.4 - 1.7	0.087	0.02 - 0.3
>0.045454545	95.00	86.1 - 99.0	41.35	36.1 - 46.8	1.62	1.5 - 1.8	0.12	0.04 - 0.4
>0.060606061	95.00	86.1 - 99.0	46.04	40.7 - 51.5	1.76	1.6 - 2.0	0.11	0.04 - 0.3
>0.075757576	95.00	86.1 - 99.0	49.85	44.4 - 55.3	1.89	1.7 - 2.1	0.10	0.03 - 0.3
>0.090909091	95.00	86.1 - 99.0	53.67	48.2 - 59.1	2.05	1.8 - 2.3	0.093	0.03 - 0.3
>0.106060606	95.00	86.1 - 99.0	56.89	51.4 - 62.2	2.20	1.9 - 2.5	0.088	0.03 - 0.3
>0.121212121	91.67	81.6 - 97.2	61.88	56.5 - 67.1	2.40	2.1 - 2.8	0.13	0.06 - 0.3
>0.136363636	91.67	81.6 - 97.2	65.40	60.1 - 70.4	2.65	2.2 - 3.1	0.13	0.05 - 0.3
>0.151515152	86.67	75.4 - 94.1	68.62	63.4 - 73.5	2.76	2.3 - 3.3	0.19	0.1 - 0.4
>0.166666667	83.33	71.5 - 91.7	71.85	66.8 - 76.6	2.96	2.4 - 3.6	0.23	0.1 - 0.4
>0.181818182	83.33	71.5 - 91.7	75.07	70.1 - 79.6	3.34	2.7 - 4.1	0.22	0.1 - 0.4
>0.196969697	78.33	65.8 - 87.9	76.83	72.0 - 81.2	3.38	2.7 - 4.3	0.28	0.2 - 0.5
>0.212121212	76.67	64.0 - 86.6	79.47	74.8 - 83.6	3.73	2.9 - 4.8	0.29	0.2 - 0.5
>0.227272727	75.00	62.1 - 85.3	81.23	76.7 - 85.2	4.00	3.1 - 5.2	0.31	0.2 - 0.5
>0.242424242	70.00	56.8 - 81.2	86.22	82.1 - 89.7	5.08	3.7 - 6.9	0.35	0.2 - 0.5
>0.257575758	70.00	56.8 - 81.2	88.56	84.7 - 91.7	6.12	4.4 - 8.6	0.34	0.2 - 0.5
>0.272727273	61.67	48.2 - 73.9	90.32	86.7 - 93.2	6.37	4.4 - 9.3	0.42	0.3 - 0.6
>0.287878788	51.67	38.4 - 64.8	91.20	87.7 - 94.0	5.87	3.9 - 8.9	0.53	0.4 - 0.7
>0.303030303	41.67	29.1 - 55.1	92.08	88.7 - 94.7	5.26	3.3 - 8.4	0.63	0.5 - 0.8
>0.318181818	40.00	27.6 - 53.5	93.26	90.1 - 95.7	5.93	3.6 - 9.8	0.64	0.5 - 0.8
>0.333333333	33.33	21.7 - 46.7	94.43	91.4 - 96.6	5.98	3.4 - 10.5	0.71	0.6 - 0.8
>0.348484848	23.33	13.4 - 36.0	95.89	93.2 - 97.7	5.68	2.9 - 11.3	0.80	0.7 - 0.9
>0.363636364	21.67	12.1 - 34.2	96.48	93.9 - 98.2	6.16	3.0 - 12.8	0.81	0.7 - 0.9
>0.378787879	18.33	9.5 - 30.4	97.07	94.7 - 98.6	6.25	2.8 - 14.1	0.84	0.7 - 0.9
>0.393939394	16.67	8.3 - 28.5	97.36	95.0 - 98.8	6.31	2.7 - 14.9	0.86	0.8 - 1.0
>0.409090909	11.67	4.8 - 22.6	97.95	95.8 - 99.2	5.68	2.1 - 15.6	0.90	0.8 - 1.0
>0.439393939	11.67	4.8 - 22.6	98.53	96.6 - 99.5	7.96	2.6 - 24.2	0.90	0.8 - 1.0
>0.454545455	10.00	3.8 - 20.5	98.53	96.6 - 99.5	6.82	2.1 - 21.6	0.91	0.8 - 1.0
>0.46969697	8.33	2.8 - 18.4	99.12	97.5 - 99.8	9.47	2.3 - 38.6	0.92	0.9 - 1.0
>0.484848485	5.00	1.0 - 13.9	99.12	97.5 - 99.8	5.68	1.2 - 27.5	0.96	0.9 - 1.0
>0.5	5.00	1.0 - 13.9	99.41	97.9 - 99.9	8.53	1.5 - 50.0	0.96	0.9 - 1.0
>0.515151515	3.33	0.4 - 11.5	99.41	97.9 - 99.9	5.68	0.8 - 39.6	0.97	0.9 - 1.0
>0.53030303	1.67	0.04 - 8.9	99.71	98.4 - 100.0	5.68	0.4 - 89.6	0.99	1.0 - 1.0
>0.590909091	1.67	0.04 - 8.9	100.00	98.9 - 100.0			0.98	1.0 - 1.0
>0.606060606	0.00	0.0 - 6.0	100.00	98.9 - 100.0			1.00	1.0 - 1.0
