# Supplementary material for: A Frailty Index based on clinical data to quantify mortality risk in dogs
Source: Sci Rep. 2019 Nov 14;9:16749. doi: 10.1038/s41598-019-52585-9 (PMC6856105; doi:10.1038/s41598-019-52585-9)
Supplement: Supplementary file 3 — Complete results of the survival analysis [file 41598_2019_52585_MOESM3_ESM.pdf]

A Frailty Index based on clinical data to quantify mortality risk in dogs

Tommaso Banzato[1*], Giovanni Franzo[1], Roberta Di Maggio[1], Elisa Nicoletto[1], Silvia Burti[1], Matteo Cesari[2,3], Marco Canevelli[4]

Complete results of the survival analysis

Model	Explanatory variables	Regression coefficients	Hazzard ratio	Standard error of the coefficent	z (Wald statistic value)	P-value
**FI**	Group0.2-0.4	2.22	9.21	0.41	5.298	<0.001
	Group>0.6	2.89	18.06	0.51	5.584	<0.001
**FI+Age**	Group0.2-0.4	2.09	8.09	0.43	4.755	<0.001
	Group>0.6	2.66	14.41	0.56	4.721	<0.001
	Age	0.04	1.04	0.05	0.948	0.343
**FI+Gender**	Group0.2-0.4	2.22	9.26	0.41	5.310	<0.001
	Group>0.6	2.89	18.04	0.51	5.582	<0.001
	Gender	0.16	1.17	0.33	0.476	0.634
**FI+BCS Category**	Group0.2-0.4	2.18	8.87	0.42	4.755	<0.001
	Group>0.6	2.81	16.69	0.39	-1.519	<0.001
	Normal BCS	-0.59	0.55	0.09	-1.776	0.0757
	Overweight	-0.95	0.38	0.48	-1.967	0.0492
**FI+Size Category**	Group0.2-0.4	2.18	8.91	0.42	5.207	<0.001
	Group>0.6	2.81	0.52	0.39	5.402	<0.001
	SizeClassM	0.98	2.66	0.46	2.123	0.033
	SizeClassL	0.87	2.40	0.55	1.570	0.116
**FI+Size Category+BCS Category**	Group0.2-0.4	2.09	8.14	0.43	4.821	<0.001
	Group>0.6	2.76	15.80	0.55	4.968	<0.001
	Normal BCS	-0.65	0.51	0.40	-1.635	0.1020
	Overweight	0.33	2.40	0.48	-2.228	0.0259
	SizeClassM	1.05	2.87	0.46	2.228	0.0225
	SizeClassL	1.04	2.85	0.56	1.854	0.0638

Table reporting the statistics of different Cox proportional hazards regression models combining FI with all other considered variables of interest.

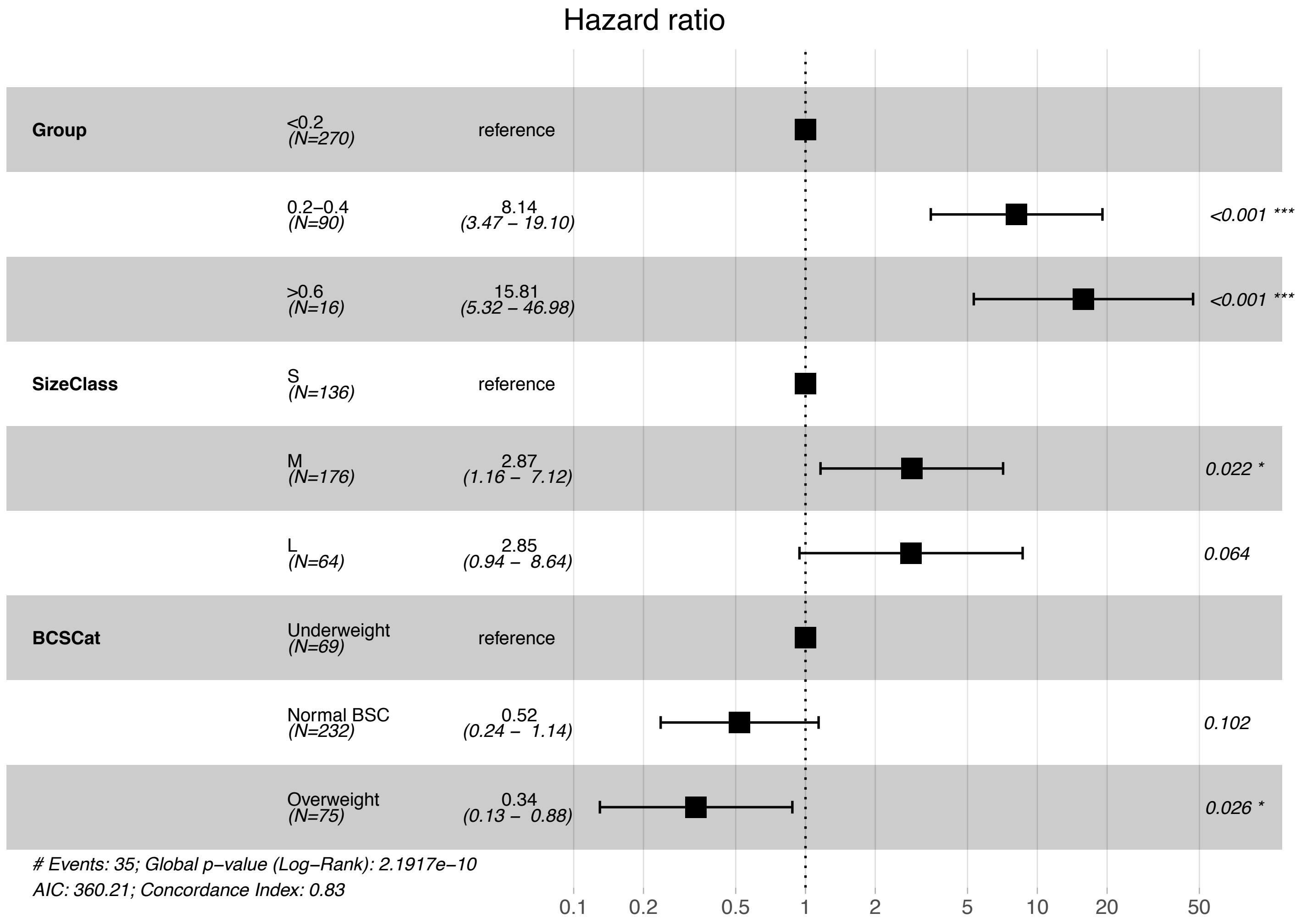

Forest Plot reporting the HR compared to the reference level and the relative 95% confidence intervals for all the explanatory variables included in the model.

Group = FI categories; SizeClass= Dog Size Category; BCSCat= Body Condition score Category.
